# Relationship between behavioral-related variables and dental caries prevalence among university students: a cross-sectional study

**DOI:** 10.3389/fpubh.2025.1683170

**Published:** 2025-12-16

**Authors:** Jiaxin Chen, Xiaoxiao Chen, Chunmiao Wang, Deqiang Zhao, Yanfeng Zhang

**Affiliations:** National Physical Fitness and Public Fitness Research Center, China Institute of Sport Science, General Administration of Sport of China, Beijing, China

**Keywords:** behavioral-related variables, Body Mass Index, dental caries, physical activity, university students

## Abstract

**Objectives:**

To examine the association between behavioral-related variables (physical activity, BMI, dental check-ups) and dental caries prevalence among university students.

**Methods:**

A cross-sectional study was conducted among 2,322 university students using WHO oral examination criteria and a standardized behavioral questionnaire. Multivariate logistic regression was performed adjusting for sex to estimate associations between PA, BMI, dental check-up behaviors and dental caries.

**Results:**

The overall caries detection rate among college students was 35.1%. The rate was significantly higher in females (43.1%) than in males (24.1%, *p* < 0.01). Binary logistic regression revealed that female gender (odds ratio [OR] = 2.30, 95% confidence interval [CI]: 1.90–2.79) was a risk factor for dental caries. Protective factors included regular dental examinations (OR = 0.56, 95% CI: 0.46–0.69), moderate-intensity physical exercise ≥30 min per week (1–2 times/week: OR = 0.47; 3–4 times/week: OR = 0.32; ≥5 times/week: OR = 0.62), and normal body mass index (BMI; OR = 0.79, 95% CI: 0.62–0.90).

**Conclusion:**

Caries prevention strategies for college students should incorporate gender-specific approaches, emphasize regular dental examinations, and promote moderate physical exercise to reduce caries risk and support lifelong oral health management.

## 1 Introduction

Dental caries remains one of the most prevalent and persistent oral diseases worldwide, affecting approximately 2.5 billion people and posing a considerable public health challenge across all age groups (1–3). Beyond its local consequences such as pain, tooth destruction, and impaired masticatory function, dental caries has been increasingly linked to a range of systemic health conditions, including cardiometabolic diseases, respiratory illness, and chronic inflammatory disorders (4, 5). Given its multifactorial etiology and lifelong health implications (6), understanding modifiable determinants of dental caries, particularly among young adults establishing long-term behavioral patterns, is of growing importance.

University students represent a unique transitional population who often experience substantial lifestyle changes, including shifts in dietary habits, reductions in physical activity, irregular sleep patterns, and decreased healthcare utilization (7, 8). These behavioral transitions may influence oral health outcomes, yet research on dental caries among young adults remains limited compared with studies focusing on children and adolescents. Existing literature highlights the importance of oral hygiene behaviors and dietary sugar intake (9), but less attention has been given to broader behavioral-related variables such as physical activity (PA) (10), body weight status, and preventive dental care practices (11). Although PA and BMI are well-established predictors of general health, their potential associations with dental caries in young adults have been insufficiently explored. A small but emerging body of evidence suggests that PA may contribute to oral health by influencing salivary function, metabolic regulation, and overall health behaviors (12, 13), while BMI has shown inconsistent relationships with caries across different populations (14, 15). These gaps underscore the need for more comprehensive analyses addressing behavioral determinants of dental caries in this age group.

In Macao, regular population-level health and fitness surveillance provides a unique opportunity to examine oral health within a broader behavioral context. However, despite the availability of such data, investigations specifically targeting the behavioral determinants of dental caries among university students remain scarce. The lack of evidence limits the ability of policymakers and health providers to design integrated health promotion strategies that address both oral and general health behaviors.

Therefore, the present study aims to address this gap by analyzing the association between behavioral-related variables, including physical activity, BMI, and dental care behaviors, and dental caries prevalence among university students. By integrating oral health assessment with behavioral and lifestyle indicators, this study seeks to provide new empirical evidence to support multidimensional caries prevention strategies tailored to young adults.

## 2 Materials and methods

### 2.1 Study population

This cross-sectional study was based on data from the 2020 Macao Citizen Physical Fitness Monitoring program. A total of 2,322 university students aged 17–22 years were included. All participants completed standardized oral examinations and behavioral questionnaires. The monitoring project was approved by the Ethics Committee of the China Institute of Sport Science (Approval No. CISSLA20190607), and written informed consent was obtained from all participants.

### 2.2 Dental caries assessment

Dental caries status was evaluated according to the World Health Organization (WHO) Oral Health Surveys: Basic Methods (4th edition) (16). Examinations were conducted by qualified dental professionals using portable equipment, including disposable mouth mirrors and WHO probes. Caries experience was recorded dichotomously (presence/absence) based on visual–tactile examination.

### 2.3 Behavioral-related variables

#### 2.3.1 Physical activity (PA)

PA information was collected using the standardized surveillance questionnaire. Moderate-intensity PA was defined in accordance with WHO guidelines (17), as activities that elevate heart rate to approximately 60–70% of the age-predicted maximum or correspond to a perceived exertion level where conversation is possible but singing is difficult. Weekly PA frequency (≥30 min per bout) was categorized as:

- 0 times/week.
- 1–2 times/week.
- 3–4 times/week.
- ≥ 5 times/week.

#### 2.3.2 Body mass index (BMI)

BMI was calculated as weight (kg) divided by height squared (m^2^). Classification followed international criteria (18):

- Underweight: <18.5.
- Normal weight: 18.5–24.9.
- Overweight: 25.0–29.9.
- Obese: ≥30.

#### 2.3.3 Dental check-up behavior

Regular dental check-up was defined as undergoing preventive oral examinations at least once per year. Students reporting no routine dental visits were categorized as “irregular.”

### 2.4 Quality control

All examiners were trained using a unified protocol prior to data collection. A subset of 5% of participants underwent repeat oral examinations to assess intra-examiner reliability. Double data entry procedures were used, and inconsistencies were resolved by checking original records.

### 2.5 Statistical analysis

All statistical analyses were conducted using SPSS version 27.0. Categorical variables were presented as frequencies and percentages. Between-group differences were assessed using chi-square tests.

Variables showing a statistical association with dental caries in univariate analyses (*p* < 0.05) were entered into multivariate logistic regression models. Sex was included as an *a priori* covariate due to its known biological and behavioral relevance to caries. Age was not incorporated because the narrow distribution (17–22 years) limited its meaningful contribution to model variability.

Adjusted odds ratios (ORs) and 95% confidence intervals (CIs) were calculated. Model fit was assessed using the Hosmer–Lemeshow goodness-of-fit test. Multicollinearity was examined using variance inflation factors (VIFs), all of which were <2, indicating no significant collinearity. A two-sided significance level of *α* = 0.05 was used for all analyses.

## 3 Results

### 3.1 Prevalence of dental caries

Among the 2,322 university students included in the analysis, the overall prevalence of dental caries was 35.1%. Female students demonstrated a significantly higher prevalence (43.1%) compared with male students (24.1%) (χ^2^ = 89.9, *p* < 0.01).

### 3.2 Univariate analysis of influencing factors for dental caries among university students

Using the presence of dental caries as the dependent variable (No = 0, Yes = 1), univariate analysis (χ^2^ test) was performed to examine the association with potential independent variables, including sex, physical activity levels, dental care habits.

The results indicated that sex, regular dental check-ups, frequency of moderate-intensity physical activity lasting ≥30 min per week, and body mass index (BMI) were significantly associated with the prevalence of dental caries among the university students (all *p* < 0.01). See Table 1.

**Table 1:** Univariate analysis of factors influencing dental caries prevalence (*n* = 2,322).

Group	Total *n*	With caries *n* (%)	Without caries *n* (%)	χ^2^ value	*P* value
Sex				93.4	<0.01
Male	972	234 (24.1)	738 (75.9)		
Female	1,350	582 (43.1)	768 (56.9)		
Regular dental check-ups				60.1	<0.01
No	1,218	516 (42.4)	702 (57.6)		
Yes	1,104	300 (27.2)	804 (72.8)		
Moderate PA ≥ 30 min/week				21.0	<0.01
0 times	570	270 (47.4)	300 (52.6)		
1–2 times	1,128	354 (31.4)	774 (68.6)		
3–4 times	414	108 (26.1)	306 (73.9)		
≥5 times	210	84 (40.0)	126 (60.0)		
Body mass index (BMI)				3.9	<0.01
Underweight	402	168 (41.8)	234 (58.2)		
Normal weight	1,512	522 (34.5)	990 (65.5)		
Overweight	324	102 (31.5)	222 (68.5)		
Obese	84	24 (28.6)	60 (71.4)		

### 3.3 Binary logistic regression analysis of factors influencing dental caries among university students

Using the presence of dental caries (No = 0, Yes = 1) as the dependent variable, variables identified as statistically significant in the univariate analysis were entered into a binary logistic regression model (Table 2).

**Table 2:** Results of binary logistic regression analysis for factors influencing dental caries (*n* = 2,322).

Independent variable	*B*	S.E.	Wald	OR (95% CI)	*P* value
Sex					
Male(Ref)	-	-	-	1.00	-
Female	0.83	0.10	71.91	2.30 (1.90 to 2.79)	<0.01
Regular dental check-ups					
No(Ref)	-	-	-	1.00	-
Yes	−0.59	0.09	39.14	0.56 (0.46 to 0.69)	<0.01
Moderate PA ≥ 30 min/week					
0 times(Ref)	-	-	-	1.00	-
1–2 times	−0.75	0.11	45.93	0.47 (0.38 to 0.59)	<0.01
3–4 times	−1.14	0.15	60.90	0.32 (0.24 to 0.43)	<0.01
≥5 times	−0.48	0.17	7.82	0.62 (0.44 to 0.87)	<0.01
Body mass index (BMI)					
Underweight (Ref)	-	-	-	1.00	-
Normal weight	−0.24	0.12	3.91	0.79 (0.62 to 0.90)	<0.05
Overweight	−0.14	0.16	0.75	0.87 (0.63 to 1.20)	0.39
Obese	−0.51	0.27	3.51	0.60 (0.35 to 1.02)	0.06

*B*, regression coefficient; S.E., standard error; Wald, Wald statistic; OR, odds ratio; CI, confidence interval; Ref, reference category.

The binary logistic regression results demonstrated that sex, regular dental check-ups, body mass index (BMI), and the frequency of moderate-intensity physical activity (≥30 min per session) per week were statistically significant independent predictors of dental caries risk among university students (all *p* < 0.05). These findings indicate that sex differences, oral health behaviors (dental check-ups), BMI status, and physical activity habits are significant influencing factors for dental caries in this population.

## 4 Discussion

This study examined the prevalence of dental caries and its behavioral determinants among university students using population-based surveillance data. Several key findings emerged. First, female students showed significantly higher odds of dental caries than males. Second, regular dental check-ups and more frequent engagement in moderate-intensity physical activity were strongly associated with reduced caries risk, demonstrating clear dose–response patterns. Third, BMI showed a modest but noteworthy association, with underweight students experiencing the highest caries prevalence. Together, these findings highlight the importance of behavioral-related variables in shaping oral health outcomes during young adulthood.

The substantially higher caries prevalence observed among female students is consistent with existing national and international studies (19, 20). Biological, behavioral, and sociocultural mechanisms may contribute to this disparity. Females typically experience earlier tooth eruption, thus extending the cumulative exposure time to cariogenic environments. Fluctuations in estrogen levels during adolescence and early adulthood may influence salivary flow and composition, indirectly affecting caries susceptibility (21–23). Behaviorally, female students may engage more frequently in snacking or consumption of sugary foods, although dietary variables were not captured in this dataset (22). These mechanisms may interact to amplify caries risk, underscoring the need for targeted oral health interventions for female university students.

Regular dental check-ups were a strong protective factor, reducing caries risk by nearly half. This finding aligns with previous work demonstrating that preventive dental attendance allows earlier detection of lesions, application of fluoride, and reinforcement of healthy behaviors (11). However, only about half of the surveyed students reported receiving regular dental examinations, which is considerably lower than coverage in many high-income countries (e.g., >80% in the Netherlands) (24). Young adults often adopt a symptom-driven approach to dental care (“problem-based attendance”), influenced by perceptions of cost, inconvenience, or underestimation of caries severity (25). Strengthening preventive dental education at universities and promoting affordable, accessible oral health services could substantially improve early detection and long-term oral health outcomes.

A key contribution of this study is the identification of a significant and graded dose–response association between PA frequency and reduced caries risk. While PA is well-established in systemic disease prevention, its potential benefits for oral health have been less frequently explored. Several mechanisms may explain the observed protective role.

First, regular PA enhances metabolic regulation and improves insulin sensitivity, reducing the frequency and duration of hyperglycemic states that promote acidogenic bacterial activity (26, 27). Second, PA stimulates salivary secretion and increases salivary pH, thereby enhancing buffering capacity and contributing to an environment less conducive to demineralization (28–30). Third, individuals who maintain regular PA routines often demonstrate healthier behavioral profiles, such as reduced sugary beverage consumption, better sleep patterns, and greater health consciousness, which may indirectly lower caries risk (31, 32). Evidence from Finnish conscripts supports these synergistic behaviors, reporting lower smoking rates and healthier dietary patterns among physically active young adults (10). The consistency between our findings and emerging literature highlights PA as a promising behavioral marker and potential leverage point for integrated oral health promotion strategies.

BMI showed a modest association with caries, with underweight students exhibiting the highest risk. This pattern has been reported in some international cohorts (33–35) and may be mediated by compromised nutritional intake, including insufficient protein, calcium, or micronutrients needed for enamel mineralization and immune competence (36, 37). Although overweight and obese groups displayed non-significant trends toward lower risk, these relationships remain complex and may vary across populations due to differing diet patterns, salivary characteristics, or health behaviors. The inconsistent directionality observed in previous research suggests that BMI alone cannot fully explain caries variation but may interact with other behavioral and metabolic factors.

The findings of this study underscore the need to integrate oral health promotion within broader health behavior frameworks targeting university students. Interventions combining regular dental check-ups with PA promotion may simultaneously benefit oral and general health. University health centers could incorporate oral health assessments into routine student check-ups and collaborate with campus fitness programs to reinforce multidimensional health behaviors. Given the higher risk among female students, gender-specific strategies, such as tailored education addressing diet, stress, and hormonal influences, may also be warranted.

This study has limitations. First, the sample was drawn exclusively from university students in Macao, which may limit the regional and socioeconomic generalizability of the findings. Second, as a cross-sectional study, it cannot establish causal relationships, particularly between physical activity and caries incidence. Third, dietary sugar intake, a well-recognized determinant of dental caries, was not assessed in this project because the surveillance system was primarily designed for physical fitness monitoring. Fourth, PA and dental care behaviors were self-reported, which may be subject to recall or social desirability bias. Furthermore, caries status was recorded only as “presence or absence” without DMF/DMFT index scoring, which restricted the ability to quantify disease severity.

Future research should expand the sample scope, incorporate standardized caries indices such as DMFT, include dietary and lifestyle assessments, and employ prospective or interventional designs to enhance the scientific robustness and generalizability of the conclusions.

## 5 Conclusion

Physical activity and regular dental check-ups are significant protective factors against dental caries among university students, while female sex remains a strong risk factor. These findings support integrated health promotion strategies combining oral health and behavioral interventions.

## Data Availability

Due to national confidentiality regulations, the date set analyzed in this study has not been publicly provided. Reasonable requests can be communicated with the corresponding author via email.

## References

[1] Bawaskar HS Bawaskar PH. Oral diseases: a global public health challenge. Lancet. (2020) 395:185–6. doi: 10.1016/S0140-6736(19)33016-8, 31954454

[2] Issrani R Reddy J Bader AK Albalawi RFH Alserhani EDM Alruwaili DSR . Exploring an association between body mass index and Oral health-a scoping review. Diagnostics. (2023) 13:902. doi: 10.3390/diagnostics13050902, 36900046 PMC10000970

[3] Das A Menon I Kumar G Singh A Jha K. Impact of physical activity on oral health: a systematic review. J Family Med Prim Care. (2025) 14:1161–8. doi: 10.4103/jfmpc.jfmpc_1124_24, 40396113 PMC12088580

[4] Altas ZM Sezerol MA. Prevalence and associated factors of dental caries in Syrian immigrant children aged 6-12 years. Children. (2023) 10:1000. doi: 10.3390/children10061000, 37371232 PMC10297169

[5] Kitsaras G Goodwin M Kelly MP Pretty IA. Bedtime Oral hygiene Behaviours, dietary habits and children's dental health. Children. (2021) 8:416. doi: 10.3390/children8050416, 34069504 PMC8160840

[6] Kashbour W Gupta P Worthington HV Boyers D. Pit and fissure sealants versus fluoride varnishes for preventing dental decay in the permanent teeth of children and adolescents. Cochrane Database Syst Rev. (2020) 11:CD003067. doi: 10.1002/14651858.cd003067.pub5 33142363 PMC9308902

[7] Adoamko Gyasi P Sun B Zhou L Zhao L. Healthcare access and utilization for international students: examining sociodemographic, lifestyle, health information, and perceived barriers. Discover Public Health. (2025) 22:665. doi: 10.1186/s12982-025-01069-5

[8] Manochaiwuthikul T Chaichutchouwakul A Yunan N Winothai N Kanta P Sapbamrer R. Health literacy disparities in Thai university students: exploring differences between health science and non-health science disciplines. BMC Public Health. (2025) 25:557. doi: 10.1186/s12889-025-21761-0, 39934783 PMC11817181

[9] Cheever VJ Mohajeri A Patel K Burris RC Hung M. Impact of free sugar consumption on dental caries: a cross-sectional analysis of children in the United States. Dent J. (2025) 13:48. doi: 10.3390/dj13020048, 39996922 PMC11854531

[10] Huttunen M Kämppi A Soudunsaari A Päkkilä J Tjäderhane L Laitala M-L . The association between dental caries and physical activity, physical fitness, and background factors among Finnish male conscripts. Odontology. (2023) 111:192–200. doi: 10.1007/s10266-022-00717-5, 35612763 PMC9810556

[11] Qu X Houser SH Tian M Zhang Q Pan J Zhang W. Effects of early preventive dental visits and its associations with dental caries experience: a cross-sectional study. BMC Oral Health. (2022) 22:150. doi: 10.1186/s12903-022-02190-6, 35488264 PMC9052678

[12] Ye D Liu Y Li J Zhou J Cao J Wu Y . Competitive dynamics and balance between *Streptococcus mutans* and commensal streptococci in oral microecology. Crit Rev Microbiol. (2025) 51:532–43. doi: 10.1080/1040841X.2024.2389386, 39132685

[13] Peng X Cheng L You Y Tang C Ren B Li Y . Oral microbiota in human systematic diseases. Int J Oral Sci. (2022) 14:14. doi: 10.1038/s41368-022-00163-7, 35236828 PMC8891310

[14] Yu S-T Hua J-Y Zeng Y-H Yu S-Y Zhang Z-Y Xiao W-S . Multimorbidity patterns of dental caries and obesity/overweight among adults: a systematic review and meta-analysis. BMC Oral Health. (2025) 25:1037. doi: 10.1186/s12903-025-06371-x, 40604755 PMC12224589

[15] Bakhoda MR Haghighat Lari MM Khosravi G Khademi Z Abbasi F Milajerdi A. Childhood obesity in relation to risk of dental caries: a cumulative and dose-response systematic review and meta-analysis. BMC Oral Health. (2024) 24:966. doi: 10.1186/s12903-024-04733-5, 39164714 PMC11334321

[16] World Health Organization. Oral health surveys: Basic methods. 4th ed. Geneva: WHO (1997).

[17] World Health Organization. WHO guidelines on physical activity and sedentary behaviour. Geneva, Switzerland: World Health Organization (2020).

[18] Kotha SB Terkawi SA Mubaraki SA Saffan ADA Kotha SL Mallineni SK. Association between body mass index (BMI) and dental caries among 6-12-year-old school children. Children. (2022) 9:608. doi: 10.3390/children9050608, 35626785 PMC9139392

[19] Lukacs JR Largaespada LL. Explaining sex differences in dental caries prevalence: saliva, hormones, and "life-history" etiologies. Am J Hum Biol. (2006) 18:540–55. doi: 10.1002/ajhb.20530, 16788889

[20] Lukacs JR. Sex differences in dental caries experience: clinical evidence, complex etiology. Clin Oral Investig. (2011) 15:649–56. doi: 10.1007/s00784-010-0445-3, 20652339

[21] Ferraro M Vieira AR. Explaining gender differences in caries: a multifactorial approach to a multifactorial disease. Int J Dent. (2010) 2010:649643. doi: 10.1155/2010/649643, 20339488 PMC2840374

[22] Alkhaldi AK Alshiddi H Aljubair M Alzahrani S Alkhaldi A Al-Khalifa KS . Sex differences in Oral health and the consumption of sugary diets in a Saudi Arabian population. Patient Prefer Adherence. (2021) 15:1121–31. doi: 10.2147/PPA.S308008, 34079232 PMC8165654

[23] Ortiz S Herrman E Lyashenko C Purcell A Raslan K Khor B . Sex-specific differences in the salivary microbiome of caries-active children. J Oral Microbiol. (2019) 11:1653124. doi: 10.1080/20002297.2019.1653124, 31497256 PMC6720314

[24] Marak M Beno P Samohyl M. Association between the dental check-ups prevalence and percentage of inhabitants with all natural teeth. Iran J Public Health. (2019) 48:179–80. doi: 10.18502/ijph.v48i1.809, 30847329 PMC6401588

[25] Chen R Santo K Wong G Sohn W Spallek H Chow C . Mobile apps for dental caries prevention: systematic search and quality evaluation. JMIR Mhealth Uhealth. (2021) 9:e19958. doi: 10.2196/19958, 33439141 PMC7840287

[26] Way KL Hackett DA Baker MK Johnson NA. The effect of regular exercise on insulin sensitivity in type 2 diabetes mellitus: a systematic review and Meta-analysis. Diabetes Metab J. (2016) 40:253–71. doi: 10.4093/dmj.2016.40.4.253, 27535644 PMC4995180

[27] Silva FM Duarte-Mendes P Teixeira AM Soares CM Ferreira JP. The effects of combined exercise training on glucose metabolism and inflammatory markers in sedentary adults: a systematic review and meta-analysis. Sci Rep. (2024) 14:1936. doi: 10.1038/s41598-024-51832-y, 38253590 PMC10803738

[28] Ligtenberg AJ Brand HS van den Keijbus PA Veerman EC. The effect of physical exercise on salivary secretion of MUC5B, amylase and lysozyme. Arch Oral Biol. (2015) 60:1639–44. doi: 10.1016/j.archoralbio.2015.07.012 26351746

[29] Frese C Frese F Kuhlmann S Saure D Reljic D Staehle HJ . Effect of endurance training on dental erosion, caries, and saliva. Scand J Med Sci Sports. (2015) 25:e319–26. doi: 10.1111/sms.12266, 24917276

[30] Alghamdi SA Aljohar A Almulhim B Alassaf A Bhardwaj SS Thomas JT . Correlation between BMI and Oral health status (DMFT, PI, mSBI, and salivary 1,5-AG) among the pediatric population in Saudi Arabia: a Clinico-biochemical study. Children. (2022) 9:1017. doi: 10.3390/children9071017, 35884001 PMC9316969

[31] Christofaro DGD Werneck AO Tebar WR Lofrano-Prado MC Botero JP Cucato GG . Physical activity is associated with improved eating habits during the COVID-19 pandemic. Front Psychol. (2021) 12:664568. doi: 10.3389/fpsyg.2021.664568 33912120 PMC8071934

[32] Cruz J Llodio I Iturricastillo A Yanci J Sánchez-Díaz S Romaratezabala E. Association of Physical Activity and/or diet with sleep quality and duration in adolescents: a scoping review. Nutrients. (2024) 16:3345. doi: 10.3390/nu16193345, 39408312 PMC11478895

[33] Gudipaneni RK Albilasi RM HadiAlrewili O Alam MK Patil SR Saeed F. Association of Body Mass Index and Waist Circumference with Dental Caries and consequences of untreated dental caries among 12-to 14-year-old boys: a cross-sectional study. Int Dent J. (2021) 71:522–9. doi: 10.1016/j.identj.2021.01.009, 33622545 PMC9275107

[34] Shi R Lin CW Li S Deng LL Lin Z Xiu LC. Obesity is negatively associated with dental caries among children and adolescents in Huizhou: a cross-sectional study. BMC Oral Health. (2022) 22:76. doi: 10.1186/s12903-022-02105-5, 35300666 PMC8932162

[35] Cheng YH Liao Y Chen DY Wang Y Wu Y. Prevalence of dental caries and its association with body mass index among school-age children in Shenzhen, China. BMC Oral Health. (2019) 19:270. doi: 10.1186/s12903-019-0950-y, 31801492 PMC6894248

[36] Turton B Sullivan S Chher T Hak S Sokal-Gutierrez K Wieringa F . Caries incidence is associated with wasting among Cambodian children. J Dent Res. (2023) 102:157–63. doi: 10.1177/00220345221126713, 36217721

[37] Nitsuwat S Webster J Sarkar A Cade J. The Association of Oral Processing Factors and Nutrient Intake in community-dwelling older adults: a systematic review and Meta-analysis. Nutr Rev. (2025) 83:e762–77. doi: 10.1093/nutrit/nuae080, 38916939 PMC11819486

